# Novel Lyssaviruses Isolated from Bats in Russia

**DOI:** 10.3201/eid0912.030374

**Published:** 2003-12

**Authors:** Alexandr D. Botvinkin, Elena M. Poleschuk, Ivan V. Kuzmin, Tatyana I. Borisova, Suren V. Gazaryan, Pamela Yager, Charles E. Rupprecht

**Affiliations:** * Plague Control Research Institute of Siberia and the Far East, Irkutsk, Russia; † Research Institute for Natural Foci Infections, Omsk, Russia; ‡ Krasnodar, Russia; § Centers for Disease Control and Prevention, Atlanta, Georgia, USA

**Keywords:** lyssavirus, rabies, bat, rhabdovirus, infectious disease, Russia, Eurasia, virus, encephalitis

## Abstract

Two new rabies-related viruses were discovered in Russia during 2002. Viruses were isolated from bats in Eastern Siberia near Baikal Lake and in the western Caucasus Mountains. After preliminary antigenic and genetic characterization, we found that both viruses should be considered as new putative lyssavirus genotypes.

Rabies is an acute, fatal encephalitis caused by lyssaviruses that are perpetuated in reservoir mammals, principally certain carnivores and bats. Although the disease has been known among carnivores, such as dogs, for centuries, the paradigm of rabies in bats has been appreciated fully only over the past 50 years (*1*–*3*). Recent findings of bat lyssaviruses throughout the world have prompted a taxonomic reconsideration of the *Lyssavirus* genus, family *Rhabdoviridae*. To date, besides their occurrence in the Americas, Africa, and Australia, at least four additional bat lyssaviruses have been identified in Eurasia (*4*–*7*). One of these has been reported from Russia, a “Duvenhage-like” virus isolated from a patient who died in 1985 after being bitten by a bat at a site near the Urkrainian border (*8*). We describe the isolation and preliminary identification of two new bat lyssaviruses discovered in Russia.

## The Study

During preliminary infectious disease surveys, bats were obtained randomly at different locations by hand at roosts and from mist netting at cave entrances and at routes of nocturnal foraging. From 1979 to 2002, a total of 210 bats were collected in the Baikal Lake region, including 98 *Vespertilio murinus*, 3 *Myotis brandtii*, 55 *M. daubentonii*, 2 *M. iknnikovii*, 29 *Eptesicus nilssonii*, 22 *Plecotus auritus*, and 1 *Murina leucogaster*. In the Caucasus Mountains, 129 bats were collected during a field expedition in July 2002, including 6 *Rhinolophus ferrumequinum*, 10 *Myotis blythii*, 43 *M. daubentonii*, 4 *M. emarginatus*, 9 *Pipistrellus kuhlii*, 2 *P. pipistrellus*, 3 *Barbastella barbastellus*, 28 *Nyctalus noctula* and 24 *Miniopterus schreibersii*. After they were collected, the bats were euthanized by cervical disposition, and tissues were removed at necropsy.

In the laboratory, bat brain samples were screened as described (*9*) by the intracerebral mouse inoculation test (MIT) with 3- to 4-week-old inbred mice and by an enzyme-linked immunosorbent assay (ELISA) with polyclonal antirabies immune globulin. Brains of animals that died during MIT were subjected to the direct fluorescent antibody test (DFAT) with polyclonal antirabies fluorescein (FITC)-labeled immune globulins (Scientific-Research Veterinary Institute, Kazan, Russia) or FITC-labeled antirabies diagnostic conjugate (Centocor Inc., Malvern, PA).

If viral antigen was detected in mouse brain by the DFAT or ELISA, the agent was further characterized by antigenic typing by using panels of antinucleocapsid monoclonal antibodies (N-MAbs) of the Centers for Disease Control and Prevention (CDC, Atlanta, GA) and N-MAbs 502-2 and 422-5 of the Wistar Institute (Philadelphia, PA) as described (*10*,*11*). For genetic typing, nucleic acid was extracted from infected brains, with amplification by reverse trancription-polymerase chain reaction (RT-PCR) and direct sequencing of the PCR products performed as described (*7*). Phylogenetic analysis of limited N gene sequences (400 bp from the amino-terminus) was performed by the neighbor joining method using MEGA software, version 2.1 for 1000 bootstrap replicates (*12*). The vesicular stomatitis virus nucleoprotein gene sequence (GenBank accession no AF473864) was used as an outgroup.

From the 339 bats examined, two lyssaviruses were isolated. In Eastern Siberia, an isolate (named Irkut virus) was obtained in the town of Irkutsk. A male Greater Tube-nosed Bat (*Murina leucogaster*) entered an apartment in September 2002. The bat was captured and maintained in captivity. The bat exhibited no abnormal behavior at first. After a few days, however, the bat became sluggish, rejected food and water, and died, approximately 10 days after capture, with signs of general exhaustion and weakness. An ELISA indicated that the bat’s brain was strongly positive with antirabies immune globulin. The brain also demonstrated typical fluorescent intracytoplasmic inclusions by DFAT, with Russian (Kazan) and Centocor FITC-globulins. In the MIT, one mouse became sick and paralyzed; he was euthanized after 18 days of incubation. The mouse’s brain was strongly positive for lyssavirus antigen by DFAT. During a second MIT intracerebral passage, the incubation period varied from 9 to 18 days. Intramuscular inoculation of mice with this virus was successful also, but susceptibility was less, producing a titer difference of log 4.2 MIC LD_50_, than with the intracerebral route.

A second isolate was obtained from the Caucasus (about 100 km southeast of the town of Krasnodar). All bats collected in this survey appeared healthy. No sick bats or carcasses were found in caves or other roosts. ELISAs of 129 bat brain samples gave negative results. The MIT produced one positive result, from the brain of a male Common Bent-winged Bat (*Miniopterus schreibersi*), captured during departure for nocturnal foraging at a cave entrance, together with 23 other males of the same species. Inoculated mice became paralyzed and died 9–13 days after intracerebral inoculation. Mouse brain impressions were DFAT-positive with either Russian (Kazan) or Centocor FITC-globulins. Intramuscular inoculation of 3-week-old mice was unsuccessful with at least log 5.7 MIC LD_50_/0.05 mL. Based on the location, the virus was named West Caucasian bat virus (WCBV).

In antigenic typing, both viruses reacted with Wistar N-MAb 502-2, but only the African nonrabies lyssaviruses reacted with N-MAb 422-5 (Table). With CDC N-MAbs, the patterns obtained for Irkut virus were similar to those of Duvenhage and European bat lyssavirus, type 1 (EBLV-1), but distinguishable from both of them, whereas WCBV demonstrated unique patterns.

**Table:** Antigenic patterns of new bat virus isolates compared to other lyssaviruses by a panel of N-MAbs^a^

Virus	N-MAbs																					
	3-1	8-2	11-1	15-2	22-3	23-4	24-1	24-10	52-1	52-2	61-1	62-4	71-2	97-3	97-11	141-1	143-1	146-3	164-2	502-2	422-5	
Irkut virus	**+**	**-**	**+**	**-**	**+**	o	**-**	**+**	**+**	**+**	**-**	**-**	**-**	**-**	**-**	**+**	**-**	**-**	**-**	**+**	**-**	
WCBV	**-**	**+**	**-**	**-**	**+**	**-**	**+**	**-**	**+**	**-**	**+**	**+**	**+**	**-**	**-**	**-**	**-**	**-**	**-**	**+**	**-**	
Lagos bat virus (variant 1)^b^	**-**	**-**	**+**	**-**	**+**	**-**	**-**	**-**	**+**	**+**	**-**	**-**	**-**	**-**	**-**	**+**	**-**	**-**	**-**	**+**	**+**	
Lagos bat virus (variant 2) ^b^	**-**	**-**	**+**	**-**	**+**	**-**	**-**	**+**	**+**	**+**	**-**	**-**	**-**	**-**	**-**	**+**	**-**	**-**	**-**	**+**	**+**	
Mokola^b^	**-**	**-**	**+**	**-**	**+**	**-**	**-**	**-**	**+**	**+**	**-**	**-**	**-**	**-**	**-**	**+**	**+**	**+**	**-**	**+**	**+**	
Duvenhage virus^b^	**-**	**-**	**+**	**-**	**+**	**+**	**-**	**+**	**+**	**+**	**+**	**-**	**-**	**-**	**-**	**+**	**-**	**-**	**-**	**+**	**+**	
EBLV-1^b^	**+**	**-**	**+**	**-**	**+**	**+**	**-**	**+**	**+**	**+**	**-**	**-**	**-**	**-**	**-**	**+**	**-**	**-**	**+**	**+**	**-**	
EBLV-2^b^	**+**	**-**	**+**	**-**	**+**	**-**	**-**	**+**	**+**	**+**	**-**	**-**	**+**	**-**	**-**	**+**	**+**	**+**	**-**	**+**	**-**	
Aravan virus	**-**	**-**	**+**	**-**	**+**	**+**	**-**	**+**	**+**	**+**	**-**	**-**	**-**	**-**	**-**	**+**	**-**	**+**	**-**	**+**	**-**	
Khujand virus	o	**-**	**-**	**-**	**+**	**+**	**-**	**-**	**-**	**-**	**-**	**-**	**-**	**-**	**-**	**+**	**-**	**+**	**+**	**+**	**-**	
Rabies, Red fox (West Europe) ^b^	**+**	**+**	**+**	**+**	**+**	**+**	**-**	**+**	**+**	**+**	**+**	**-**	**+**	**+**	**+**	**+**	**-**	**+**	**+**	**+**	**-**	
Rabies, Red fox (Caucasus)	**+**	**+**	**+**	**+**	**+**	**+**	**-**	**+**	**+**	**+**	**+**	o	**-**	**+**	**+**	**+**	**-**	**+**	**+**	**+**	**-**	
Rabies, CVS	**+**	**+**	**+**	**+**	**+**	**+**	**+**	**+**	**+**	**+**	**+**	**+**	**+**	**+**	**+**	**+**	**+**	**+**	**+**	**+**	**-**	

^a^N-MAbs, antinucleocapsid monoclonal antibodies; –, absence of reaction; zero, reduced reaction with 10^x^ less diluted antibody; +, positive reaction; WCBV, West Caucasian bat virus; .EBLV, European bat lyssavirus.     ^b^Patterns obtained from Smith (*11*).

When phylogenetic analysis was performed, Irkut virus was recognized as a member of a cluster-joining lyssavirus genotypes 4 and 5 (76% bootstrap support). However, the degree of diversity did not allow us to consider it a representative of one of these genotypes (Figure). WCBV was connected to the cluster of genotypes 2 and 3, but bootstrap support of this joining was insignificant (68%), illustrating that this virus is the most divergent member of the *Lyssavirus* genus examined to date. Further analysis of the entire N and other genes should be conducted to refine the phylogenetic relationships of both these viruses.

**Figure:**
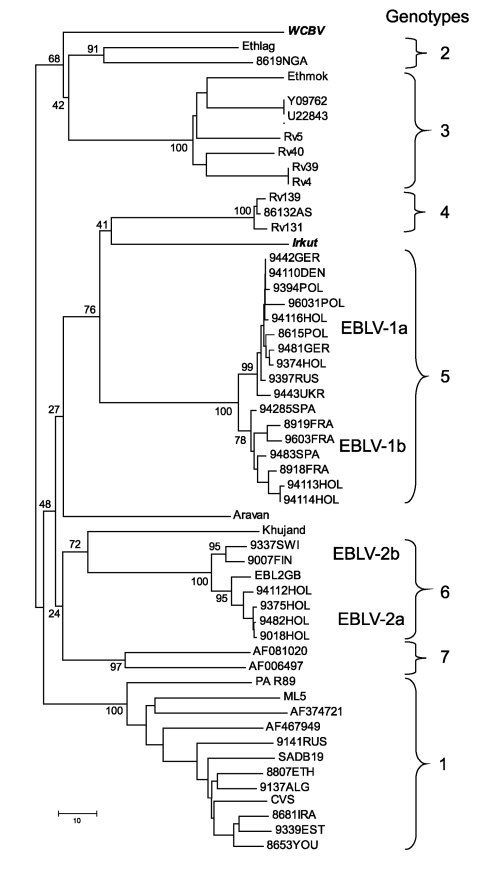
Neighbor-joining phylogenetic tree of the *Lyssavirus* genus, based on limited N gene sequences (400 bp from the amino terminus). Virus names are provided according to GenBank records, except for Ethmok and Ethlag. Subgroups “a” and “b” of EBLV-1 and EBLV-2 viruses are given according to Amengual et al. (*5*). Bootstrap values are presented for key nodes, and branch lengths are drawn to scale.

## Conclusions

Estimating the potential public health significance of these two newly recognized lyssaviruses is critical. Other bat lyssaviruses cause fatal human encephalitis, even in so-called “rabies-free” countries (*2*,*3*,*13*). Given bat mobility and the opportunity for infecting new areas quickly, no major geographic area can be considered truly free from lyssaviruses. For example, the Irkutsk Province was considered free of rabies for 35 years before the Irkut virus was isolated. Additionally, although the Caucasus had been considered as a rabies-endemic area, virus reservoirs were identified only among the canids. Public health authorities need to be aware of the potential of bats to transmit lyssaviruses and increase surveillance and public education. Attention should focus on the protective efficacy of commercially available rabies virus vaccines and immune globulins against these novel nonrabies lyssaviruses, before human infection occurs.
